# Effects of Length of Vasopressors Infusion on Mortality

**DOI:** 10.7759/cureus.5336

**Published:** 2019-08-07

**Authors:** Vikas Kumar, Shreyas Srinivas, Thuy-Hong Le, Martin Barnes, Sonia Kumar, John Redinski

**Affiliations:** 1 Internal Medicine, Donald and Barbara Zucker School of Medicine at Hofstra / Northwell, Port Jefferson, USA; 2 Internal Medicine, Kaiser Permanente Fontana, Fontana, USA; 3 Internal Medicine, Des Moines University, Des Moines, USA; 4 Critical Care, Arrowhead Regional Medical Center, Colton, USA

**Keywords:** septic shock, critical care, shock, apache-ii, mortality

## Abstract

Background

There has been a myriad of studies into the physiology of vasopressors and the survival benefit of vasopressor initiation in cases of septic shock. The aim of the Effects of Length of Vasopressors Infusion on Mortality (ELVI-Mortality) study is to observe a relationship between the length of vasopressor infusion and mortality in the intensive care unit.

Methods

This was a single-center, retrospective, matched-cohort study that collected data from Arrowhead Regional Medical Center’s electronic medical records (EMR; MediTech, MA, US) using International Classification of Diseases, Tenth Revision (ICD-10) coding and a chart reviewing the past five years. Patients were on norepinephrine, phenylephrine, or epinephrine. Two patient groups were compared. The first group encompassed those with vasopressor infusion less than 48 hours in duration, whereas the second group included those with vasopressor infusion greater than 48 hours.

Results

A total of 193 patients were diagnosed as having septic shock. Participant data were obtained for 163 patients (84.4%). Of the 106 patients who had a vasopressor duration of less than 48 hours, 32 patients (30.2%) expired. Of the 57 patients that had more than 48 hours of vasopressor infusion, 18 patients (31.6%) expired.

Conclusions

There was no statistically significant increase in mortality in patients with vasopressor infusion greater than 48 hours as compared to less than 48 hours.

## Introduction

Vasopressors can be lifesaving when applied in certain situations, but they also come with serious adverse effects, including but not limited to arrhythmias, peripheral ischemia, glucose abnormalities, organ toxicities (liver, cardiac, pulmonary, renal), and drug-drug interactions [1-2]. Multiple studies have been conducted supporting the timely initiation of fluid resuscitation and vasopressor infusion in the setting of septic shock. However, to our knowledge, there have been no studies that have looked into the relationship between the length of vasopressor infusion and overall mortality [3].

## Materials and methods

We performed a single-center, retrospective, cohort study by collecting data from Arrowhead Regional Medical Center’s electronic medical records (MediTech, MA, US) using International Classification of Diseases, Tenth Revision (ICD-10) coding and reviewing charts from 2012-2017. Patients 18 years of age or older and diagnosed with septic shock, either on admission or during hospital stay, were evaluated for the duration of vasopressor administration. Patients were on norepinephrine, phenylephrine, or epinephrine. Two patient groups were compared. The first group encompassed those with vasopressor infusion less than 48 hours of duration, whereas the second group included those with vasopressor infusion greater than 48 hours. Overall mortality by the end of hospitalization was compared between these two groups. Other types of shock and other causes of hypotension were excluded.

The variables that were addressed in this study included age, gender, source of infection, isolated organism, subsequent diagnosis of end-organ failure (multiple organ dysfunction syndrome (MODS), renal failure, or liver failure), mean arterial pressure (MAP) recorded during vasopressor therapy, acute physiology and chronic health evaluation (APACHE) score, body mass index (BMI), corticosteroid, and the use of vasopressin were evaluated (Table 1).

**Table 1:** Analysis of clinical features of the two study groups BMI: body mass index; UTI: urinary tract infection; APACHE: acute physiology and chronic health evaluation

	<48 hours	>48 hours
Total	106	57
Mortality	32	18
Percent Mortality	30.2%	31.6%
BMI	30.7 ± 9.3	29 ± 8.4
Steroid	13	49
Ethnicity:		
Caucasian	39	22
African-American	15	2
Asian	6	4
Hispanic	45	29
American Indian	1	0
Age	57.9 ± 16.1	63.2 ± 13.1
Source:		
Abdominal	26	7
Skin	17	12
UTI	29	9
Thoracic	27	25
Multiple	7	4
Multiple Organ Dysfunction Syndrome	74 (69.8%)	42 (73.7%)
Multiple Vasopressors	10	3
APACHE	14.9 ± 6.4	17.2 ± 6.7

## Results

A total of 193 patients diagnosed with septic shock were evaluated for vasopressor duration. Of those, 163 had met this study’s inclusion criteria and 30 were excluded due to exclusion criteria or insufficient documentation. A total of 106 patients had a vasopressor duration of less than 48 hours; of these, 32 patients (30.2%) expired during hospitalization. Out of the 57 patients that had more than 48 hours of vasopressor infusion, 18 patients (31.6%) expired during hospitalization.

**Figure 1:**
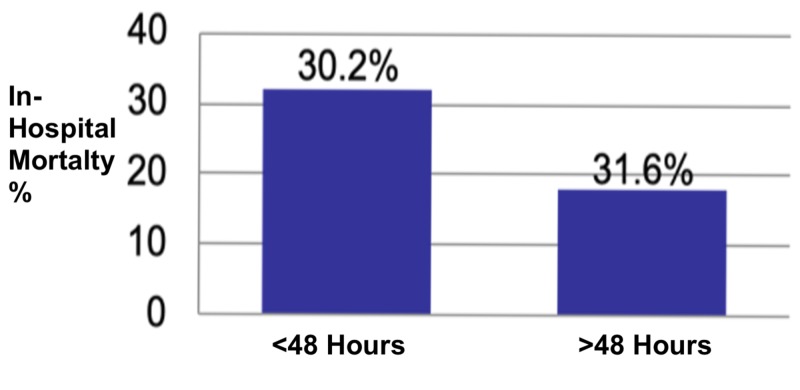
Analysis of those who expired showed no statistical difference at the 0.05 level of significance (p-value 0.8604)

A two-tailed t-test was performed on these data, demonstrating no statistical significance between the two groups on mortality (p=0.8604) (Figure 1).

## Discussion

Vasopressors are a class of intravenous drugs used to elevate the mean arterial pressure (MAP). They work through complex adrenergic receptors and dopaminergic receptors [4].

According to the Surviving Sepsis Guidelines, vasopressors are a key intervention for hemodynamic support after proper fluid resuscitation. The goal of therapy is to target a MAP of 65 mmHg [5]. Vasopressors are indicated for a decrease of >30 mmHg from baseline systolic blood pressure or MAP <60 mmHg, as either condition results in end-organ dysfunction secondary to hypoperfusion [6-7].

In our single-center, retrospective analysis of patients diagnosed with septic shock at a large county medical center, we found no statistically significant increase in mortality in patients with vasopressor infusion greater than 48 hours as compared to less than 48 hours.

We encountered numerous limitations and unexpected difficulties with our study. First, as a retrospective study, we were unable to randomize and standardize our patients. Furthermore, many patients were excluded from our study due to other causes of shock, i.e. hypovolemic and cardiogenic, which were superimposed with infection. There was also inconsistent documentation of vasopressor administration and sources of septic shock.

Further analysis could include the evaluation of vasopressor duration in more frequent intervals, e.g. hourly and mortality association. Numerous patients encountered during the chart review process had expired within a few hours of initiating vasopressor infusion. These patients expired from either being found down for a prolonged period of time, reaching advanced stages of infection, or changing the patient’s code status (i.e. withdrawal of care). This study offers some experiential insights into the design of future research on vasopressor usage in the setting of septic shock.

## Conclusions

Despite widespread use, vasopressors have not been adequately studied with regard to infusion time. In our single-center retrospective analysis, a vasopressor duration of greater than 48 hours was not associated with an increase in mortality. Though our study found no association, further investigation into vasopressor utilization and mortality benefit in the intensive care unit (ICU) is warranted.
